# Epithelial–mesenchymal plasticity is associated with transcriptional variation in regulated cell death and immune signaling programs in lung adenocarcinoma

**DOI:** 10.3389/fcell.2026.1980156

**Published:** 2026-10-20

**Authors:** Kuiyuan Liu, Jia Liu, Tiebao Meng, Jingeng Liu, Fangming Wu

**Affiliations:** 1 Zhengzhou Key Laboratory of Thoracic Oncology, Henan Medical Key Laboratory of Thoracic Oncology, Department of Thoracic Surgery, Henan Molecular Pathology and Clinical Experimental Engineering Research Center of Thoracic Disease, Advanced Medical Research Center for Prevention and Treatment of Thoracic Disease, The First Affiliated Hospital of Zhengzhou University, Lung Cancer Diagnosis and Treatment Research Institute of Zhengzhou University, Zhengzhou, Henan, China; 2 Department of Intensive Care Unit, Sun Yat-sen University Cancer Center, Guangzhou, Guangdong, China; 3 Department of Radiology, Sun Yat-sen University Cancer Center, Guangzhou, Guangdong, China; 4 Department of Interventional Radiology, Henan Provincial People’s Hospital, Zhengzhou, Henan, China

**Keywords:** epithelial–mesenchymal plasticity, lung adenocarcinoma, regulated cell death, RT-qPCR, single-cell RNA sequencing, TCGA-LUAD, transcriptional signatures

## Abstract

**Background:**

Epithelial–mesenchymal plasticity is associated with variation in regulated-cell-death (RCD) transcription and tumor–immune communication in lung adenocarcinoma (LUAD). We examined these relationships across single-cell, bulk-transcriptomic, and cell-line expression data.

**Methods:**

GSE131907 UMI counts underwent quality control, normalization, dimensionality reduction, and Leiden clustering. Epithelial and mesenchymal programs were scored against expression-matched control genes; 40th/60th-percentile state assignments were compared with median and quartile rules. CNV profiles were inferred in 300 malignant-label cells and 100 normal epithelial references. Malignant-cell count pseudobulk and separate pro-/anti-apoptosis and pro-/anti-ferroptosis scores were analyzed. TCGA-LUAD included 539 tumors and 59 normal samples; overall survival was evaluated by median-split Kaplan-Meier analysis and Cox models adjusted for age, sex, and stage. RT-qPCR compared BIRC3, CXCR4, MALAT1, and GPX4 between TGF-β1-treated (10 ng/mL, 48 h) and vehicle-treated control groups in A549 and NCI-H1975 cells.

**Results:**

The single-cell analysis retained 203,157 cells, including 24,341 malignant-label cells. Median inferred CNV burden was 3.15-fold higher in the malignant-label subset than in normal epithelial references. The malignant EMT-high fraction correlated positively with the myeloid fraction (ρ = 0.539, P = 0.012; BH-adjusted q = 0.059 across five comparisons). Tumor-versus-normal fold changes showed 91.5% direction concordance among genes significant in both platforms (ρ = 0.82). The EMT–RCD composite score was not independently associated with survival (HR = 0.973, 95% CI 0.829–1.141; P = 0.736). CXCR4 expression increased 2.43-fold in A549 and 2.12-fold in NCI-H1975 cells; BIRC3 and MALAT1 increased significantly only in A549 cells.

**Conclusion:**

LUAD EMT-associated transcriptional heterogeneity coincides with variation in death-regulatory programs and myeloid composition. CXCR4 showed a consistent expression response in both cell lines, whereas the composite transcriptional score had no independent association with overall survival. These findings prioritize CXCR4-associated cell-state biology for further investigation.

## 1 Introduction

Regulated cell death is not a single terminal event but a network of molecular programs with distinct inflammatory consequences. Apoptosis, ferroptosis, necroptosis, pyroptosis, and other forms of RCD differ in membrane integrity, metabolic requirements, execution machinery, and the signals exposed or released to the extracellular space (Galluzzi et al., 2018; Tang et al., 2019; Bedoui et al., 2020; Weinlich et al., 2017). These differences matter because dying cells can either promote resolution or initiate sterile inflammation. DAMPs such as HMGB1, extracellular ATP, and surface-exposed calreticulin can engage innate immune sensing, but their biological meaning depends on the temporal and molecular context in which they are emitted (Galluzzi et al., 2017; Galluzzi et al., 2020; Roh and Sohn, 2018; Tang et al., 2023). Thus, the clinically relevant question is not simply whether cells die, but which death state is accessible, which inflammatory signals accompany it, and whether those signals support immune clearance or chronic tissue injury.

Cancer is not conventionally classified as a chronic inflammatory disease, yet the tumor microenvironment provides a clinically important model of persistent cell stress, incomplete cell clearance, immune reprogramming, and recurrent death-survival selection. Tumor-promoting inflammation and phenotypic plasticity are now recognized as central enabling dimensions of malignancy (Hanahan, 2022). In NSCLC, malignant epithelial cells are continuously exposed to hypoxia, oxidative stress, immune pressure, and treatment, all of which can alter both cell state and RCD competence. This makes NSCLC particularly suitable for studying the interface between cell death and inflammation without equating cancer with classical autoimmune or inflammatory disorders.

EMT is a dynamic spectrum rather than a binary switch (Nieto et al., 2016). Hybrid and mesenchymal states can promote migration, stemness, immune escape, and treatment tolerance, while also changing the metabolic dependencies of malignant cells (Gu et al., 2023). Importantly, highly mesenchymal therapy-resistant states are often dependent on GPX4-mediated lipid peroxide detoxification and therefore acquire a specific relationship with ferroptosis (Jiang et al., 2021; Viswanathan et al., 2017; Hangauer et al., 2017). EMT may also interact with apoptosis-regulatory proteins, hypoxia-responsive chemokine pathways, and epigenetic regulators, raising the possibility that it coordinates several death-related programs at once rather than affecting a single pathway.

The inflammatory output of these state transitions is equally important. ICD is a functional phenotype in which dying cells generate adaptive immune responses through coordinated antigenicity and adjuvanticity; no individual DAMP measurement is sufficient to establish ICD on its own (Galluzzi et al., 2017; Galluzzi et al., 2020). Likewise, circulating IL-1β or IL-18 may reflect inflammasome-associated signaling but cannot by themselves prove pyroptotic execution, which requires evidence of gasdermin activation and membrane permeabilization (Broz et al., 2020; Yu et al., 2021). A rigorous translational framework therefore needs to distinguish pathway-associated signatures from direct evidence of completed cell death.

Liquid-biopsy studies provide a broader translational context for connecting tumor-cell states with accessible molecular measurements (Smit and Pantel, 2024; Alix-Panabières and Pantel, 2025). The present analysis focuses on tissue transcriptomes and cell-line gene expression.

We therefore examine GSE131907 single-cell and TCGA-LUAD bulk expression data alongside RT-qPCR analysis of A549 and NCI-H1975 cells BIRC3, CXCR4, MALAT1, and GPX4 were selected to represent apoptosis regulation, chemokine signaling, RNA-mediated regulation, and ferroptosis defense, respectively (Viswanathan et al., 2017; Hangauer et al., 2017; Kim et al., 2021; Li et al., 2023; Amodio et al., 2018; Ren et al., 2016;Flavahan et al., 2017; Salvesen and Duckett, 2002; Fulda, 2017). The analysis tests the association of transcriptional programs with cell state and the tumor microenvironment, followed by an assessment of their relationship with overall survival.

## 2 Methods

### 2.1 Data sources

The GSE131907 discovery cohort comprises 208,506 cells from 58 LUAD-associated samples and 44 patients, covering primary tumors, normal lung, normal lymph nodes, lymph-node and brain metastases, bronchoscopy/EBUS specimens, and pleural effusions (Kim et al., 2020). The deposited UMI count matrix, cell-annotation table, and sample feature summary were used to construct the analysis dataset. All 58 samples were retained in the atlas. Sample identifiers, patient identifiers, and tissue origins are listed in Supplementary Table S1.

TCGA-LUAD bulk data comprised 539 primary tumors and 59 solid-tissue normal samples after exclusion of two recurrent-tumor samples. These samples were used for tumor-versus-normal expression analysis. GEO single-cell and TCGA bulk analyses were integrated by gene-level comparison of the corresponding expression contrasts. Supplementary Table S2 lists all 600 input barcodes, the 598 expression-analysis inclusions, the two recurrent-tumor exclusions, and survival-analysis inclusion status. The 539 primary-tumor specimens represented 516 patients; expression comparisons used specimens, whereas survival analyses used one selected specimen per patient.

### 2.2 Single-cell preprocessing and quality control

Library quality metrics were calculated from the full UMI matrix. Cells with 500–8,000 detected genes and a mitochondrial UMI fraction below 20% were retained. Counts were normalized to 10,000 per cell and transformed with log1p. The analysis selected 3,000 highly variable genes using the cell_ranger method with sample as the batch key. Highly variable features were scaled with clipping at 10, followed by 50 principal components, a 15-nearest-neighbor graph, UMAP, and Leiden clustering at resolution 1.0. Full-gene log-normalized expression was retained for program scoring. PCA, neighbor-graph, UMAP, and clustering steps were implemented in the analysis scripts; canonical lineage markers were examined alongside the GEO annotations (Kim et al., 2020; Wolf et al., 2018). Pre-filter QC denotes the deposited UMI matrix before the filters applied in this study. Its maximum observed mitochondrial fraction was 20%, so the ECDF reaches 1 at that boundary.

### 2.3 Malignant-cell definition and EMT state assignment

Malignant-cell candidates were identified from the GSE131907 malignant-cell annotation and evaluated with epithelial-marker expression and an independently computed expression-based CNV profile. Normal epithelial cells from normal-lung or normal-lymph-node samples formed the reference pool. Using random seed 1, 300 malignant-label cells and 100 normal epithelial cells were sampled for infercnvpy 0.6.1 analysis (infercnvpy developers, 2026). The input contained 18,483 genes with genomic coordinates; the analysis used a 50-gene window, 25-gene step, log-normalized expression, and normal epithelial cells as the reference. Per-cell CNV burden was the mean absolute inferred signal across genomic windows. The full 24,341-cell malignant compartment was defined by the annotation, with CNV-burden comparison reported for the 400-cell subset (Supplementary Table S7). Autosomal gene coordinates were matched by gene symbol; the expression-difference clip was 3 and the dynamic noise threshold was 1.5 standard deviations.

Epithelial (E) genes were EPCAM, CDH1, KRT8, KRT18, and KRT19; mesenchymal (M) genes were VIM, FN1, CDH2, SNAI1, SNAI2, ZEB1, ZEB2, and TWIST1. For a gene set G, score_genes calculated S (G) = mean log-normalized expression of G minus mean expression of a matched control-gene set (Wolf et al., 2018). Control genes were sampled from 25 expression bins with ctrl_size = 50 and random_state = 0. Primary E/M scores used the full QC-passing atlas as the expression background, and thresholds were calculated within the malignant compartment. Epithelial-like cells had E ≥ Q60(E) and M < Q40(M); hybrid E/M cells had E ≥ Q60(E) and M ≥ Q60(M); mesenchymal-like cells had M ≥ Q60(M) and E < Q40(E). All remaining cells formed the intermediate category, including cells within the central score bands and cells with low values of both programs. The central 40th–60th percentile interval was used as a buffer between low and high scores. Its effect was evaluated using median and 25th/75th-percentile alternatives with the same E/M scores. This two-program representation follows the multi-state view of epithelial–mesenchymal plasticity (Nieto et al., 2016; Yang et al., 2020). These cutoffs define within-cohort transcriptional categories. Under the median rule, Q40 and Q60 were both replaced by Q50; under the quartile rule, they were replaced by Q25 and Q75, respectively. The same classification inequalities were then applied (Supplementary Table S4).

### 2.4 RCD and immune signatures

RCD gene sets were separated according to regulatory direction. The pro-apoptosis set comprised BAX, CASP3, CASP7, APAF1, and CYCS; the anti-apoptosis set comprised BIRC3, BIRC2, BCL2, BCL2L1, and XIAP (Galluzzi et al., 2018; Tang et al., 2019; Bedoui et al., 2020; Salvesen and Duckett, 2002; Fulda, 2017). The pro-ferroptosis set comprised ACSL4 and TFRC, whereas SLC7A11 and GPX4 formed the anti-ferroptosis set (Jiang et al., 2021; Viswanathan et al., 2017; Hangauer et al., 2017). PTGS2 was examined separately as a ferroptosis-associated response marker, consistent with experimental evidence that its induction can occur downstream of ferroptosis (Yang et al., 2014). Each set was scored separately in malignant cells using score_genes with 25 expression bins, ctrl_size = 50, and random_state = 0. The apoptosis and ferroptosis balance indices were defined as S (pro) − S (anti); higher values indicate a relative shift toward the corresponding pro-death transcriptional program. A difference was used because control-adjusted module scores can be zero or negative, making their direct ratio unstable. DAMP-associated genes were CALR, HSPA1A, HMGB1, HMGB2, and P2RX7; inflammasome-associated genes were NLRP3, GSDMD, CASP1, IL1B, and IL18 (Supplementary Table S5).

### 2.5 Statistical units, differential expression, and multiplicity

For differential expression, UMI counts were summed within each biological sample and cell compartment. Malignant pseudobulk analysis required at least 20 malignant cells per sample, yielding 21 samples from 21 distinct patients (Supplementary Table S3). Each patient contributed one malignant sample to this comparison. EMT-high cells were defined as the combined hybrid E/M and mesenchymal-like categories. Samples with an EMT-high cell fraction ≥0.20 were assigned to the EMT-high group (17 patients), and the remaining four patients to the EMT-low group. PyDESeq2 modeled EMT-high versus EMT-low malignant samples and malignant versus normal-epithelial samples, with group as the design factor. Genes with zero total counts were excluded; log2 fold changes, standard errors, Wald-test P values, and Benjamini–Hochberg-adjusted P values were reported for each contrast.

For the sample-level E/M and directional RCD analyses, malignant UMI counts were first summed by sample, normalized to 10,000, log1p-transformed, and then scored. Each plotted observation therefore represents a malignant-cell count pseudobulk sample. Spearman correlations quantified program associations and relationships between the malignant EMT-high fraction and five cellular fractions (T, NK, myeloid, B, and fibroblast). The five immune-composition comparisons were additionally adjusted together using the Benjamini–Hochberg procedure. Per-cell plots display expression distributions and state composition.

### 2.6 Ligand–receptor and bulk transcriptome analyses

Malignant-to-immune ligand–receptor co-expression was summarized as the geometric mean of mean log-normalized ligand expression in sender cells and mean log-normalized receptor expression in receiver cells. The curated ligand–receptor list is provided in Supplementary Table S8. For each ligand–receptor/receiver combination, the EMT-high proxy was the unweighted mean of the hybrid E/M and mesenchymal-like sender proxies; the epithelial-like proxy was subtracted to obtain the displayed difference. This quantity describes relative ligand–receptor transcript co-expression. TCGA tumor-versus-normal differential expression was analyzed with PyDESeq2 and visualized on the log2(CPM+1) scale. Cross-platform analyses compared malignant-versus-normal-epithelial pseudobulk log2 fold changes with TCGA tumor-versus-normal log2 fold changes. Direction concordance and Spearman correlation were calculated for all shared genes and for genes with BH-adjusted P < 0.05 in both datasets.

The EMT–RCD composite was defined as C = mean [z (M genes), z (BIRC3), z (CXCR4), z (GPX4)] − mean [z (E genes)], where each gene was standardized across the 539 TCGA tumors using its mean and sample standard deviation. The positive term averages 11 genes together; the negative term averages five epithelial genes. Thus, C combines mesenchymal expression with apoptosis regulation, chemokine signaling, and ferroptosis defense. Gene inclusion was based on the functions represented by these components (Nieto et al., 2016; Viswanathan et al., 2017; Hangauer et al., 2017; Kim et al., 2021; Salvesen and Duckett, 2002; Fulda, 2017), and equal weighting provided a transparent summary of their expression. MALAT1 was evaluated in the cell-line experiment and was absent from the TCGA expression matrix used for scoring. Component analyses separately calculated the EMT axis [mean z(M) − mean z(E)], apoptosis balance, ferroptosis balance, and the sum of these three indices. For the latter two balances, mean gene z-scores replaced the single-cell module scores while retaining the same pro-minus-anti definitions. The composite was evaluated as an equal-weight expression summary; the component gene literature provides the biological rationale for inclusion. Composite-to-EMT-axis correlation was calculated across the TCGA tumors to assess how strongly the combined index followed its EMT component.

### 2.7 Survival analysis

Overall survival was calculated from vital status and days to death or last follow-up for 507 patients, including 183 deaths. The composite-score median was determined in the 539-tumor expression cohort and applied to patients with available follow-up. Kaplan–Meier estimates included 95% confidence bands, and group differences were assessed with the log-rank test. Multivariable Cox models used continuous scores and adjusted for age, sex, and pathologic stage grouped as I–IV; complete-case models included 478 patients and 172 deaths. Hazard ratios represent a one-unit increase in the specified score. Score distributions across stages and vital-status groups were compared using Kruskal–Wallis and Mann–Whitney tests, respectively. Where a patient had multiple primary-tumor specimens, the first specimen in lexicographic barcode order was selected before excluding missing or negative follow-up times.

### 2.8 Experimental RT-qPCR analysis

Total RNA was isolated from A549 (ATCC CCL‐ 185; American Type Culture Collection, 2026a) and NCI‐ H 1975 (ATCC CRL‐ 5908; American Type Culture Collection, 2026b) cells using TRIzol reagent (Invitrogen, Carlsbad, CA, United States) according to the manufacturer’s instructions, and 1 μg of total RNA was reverse-transcribed into cDNA using the PrimeScript RT reagent Kit with gDNA Eraser (Takara Bio, Shiga, Japan). RT-qPCR was performed on a Bio-Rad CFX96 real-time PCR detection system using SYBR Premix Ex Taq II (Takara Bio) with GAPDH as the internal reference gene. Relative expression was calculated using the 2^−ΔΔCq method (Bustin et al., 2025; Livak and Schmittgen, 2001). Primer pairs were: BIRC3, forward 5′-CCA​AGT​GGT​TTC​CAA​GGT​GT-3′ and reverse 5′-TGG​GCT​GTC​TGA​TGT​GGA​TA-3′; CXCR4, forward 5′-GGC​CAA​GTT​CTT​AGT​TGC​TGT​ATG-3′ and reverse 5′-ACG​TTC​CAC​GGG​AAT​GGA​G-3′; MALAT1, forward 5′-TGG​GGG​AGT​TTC​GTA​CTG​AG-3′ and reverse 5′-TCT​CCA​GGA​CTT​GGC​AGT​CT-3′; GPX4, forward 5′-GCC​TTC​CCG​TGT​AAC​CAG​T-3′ and reverse 5′-GCG​AAC​TCT​TTG​ATC​TCT​TCG​T-3′; GAPDH, forward 5′-CTC​CTC​CAC​ATT​TGA​CGC​TG-3′ and reverse 5′-TCC​TCT​TGT​GCT​CTT​GCT​GG-3′. TGF‐β1 has been reported to induce EMT in A549 cells (Kasai et al., 2005), while combined TGF‐β1/TNF‐α treatment has been associated with EMT‐related gene‐expression changes in this cell line (Nurwidya et al., 2017). For EMT induction, cells were treated with recombinant human TGF-β1 (R&D Systems, Minneapolis, MN, United States; catalog no. 240-B) at 10 ng/mL for 48 h, whereas control cultures received an equal volume of vehicle (4 mM HCl containing 1 mg/mL bovine serum albumin). RT-qPCR compared BIRC3, CXCR4, MALAT1, and GPX4 expression between the two conditions. Four biological replicates were analyzed per group, with three technical replicates per biological replicate.

The RT-qPCR analysis compared expression of the four candidate genes between TGF-β1-treated (10 ng/mL, 48 h) and vehicle-treated control groups in each cell line. Technical replicates were nested within biological replicates, with biological replicates forming the unit of analysis. Relative-quantification terminology follows the RT-qPCR literature: ΔCq denotes the target-minus-reference quantification cycle, ΔΔCq denotes the difference relative to the control calibrator, and 2^−ΔΔCq expresses relative abundance when the comparative-Cq assumptions apply (Bustin et al., 2025; Livak and Schmittgen, 2001). The reported experimental fold changes were calculated from the group means of normalized expression, as specified below.

### 2.9 RT-qPCR statistical analysis

Four biological replicates were analyzed per group, with three technical replicates per biological replicate; technical replicates were not counted as independent biological replicates. For the supplied biological-replicate Cq matrix, ΔCq values were calculated as target Cq minus GAPDH Cq. Welch two-sample t tests compared ΔCq values between treatment and control groups, and P values were adjusted within each cell line across the genes with available Cq values using the Benjamini–Hochberg procedure. The raw matrix yielded A549 adjusted P values of 0.0390 (BIRC3), 0.0018 (CXCR4), 0.0056 (MALAT1), and 0.1885 (GPX4), and NCI-H1975 adjusted P values of 0.0093 (CXCR4) and 0.1696 (MALAT1). For NCI-H1975, the supplied matrix included two of the four assayed genes. BIRC3 and GPX4 Cq values were not supplied, so their significance designations remain based on the source experimental summary. The analysis presents aggregate log2 fold changes without error bars.

## 3 Results

### 3.1 Cohort architecture and quality control of the GSE131907 atlas

The discovery cohort comprised 58 samples from 44 patients spanning seven tissue origins. After quality control on the full gene panel (500–8,000 detected genes, mitochondrial fraction <20%), 203,157 of 208,506 cells (97.4%) were retained. Per-sample retention ranged from 1,011 to 5,756 cells (Figure 1A). Pre-filter library complexity showed a median of 1,103 detected genes and 3,489 UMIs per cell with a median mitochondrial fraction of 3.53% (Figure 1B); origin-specific ECDFs are shown in Figure 1C. Per-sample QC retention is shown in Figure 1D. Post-QC library depth by origin is summarized in Figure 1E, and the cellular composition of each origin in Figure 1F, where epithelial cells constituted 16.3% of primary-tumor and 8.6% of normal-lung cells.

**FIGURE 1:**
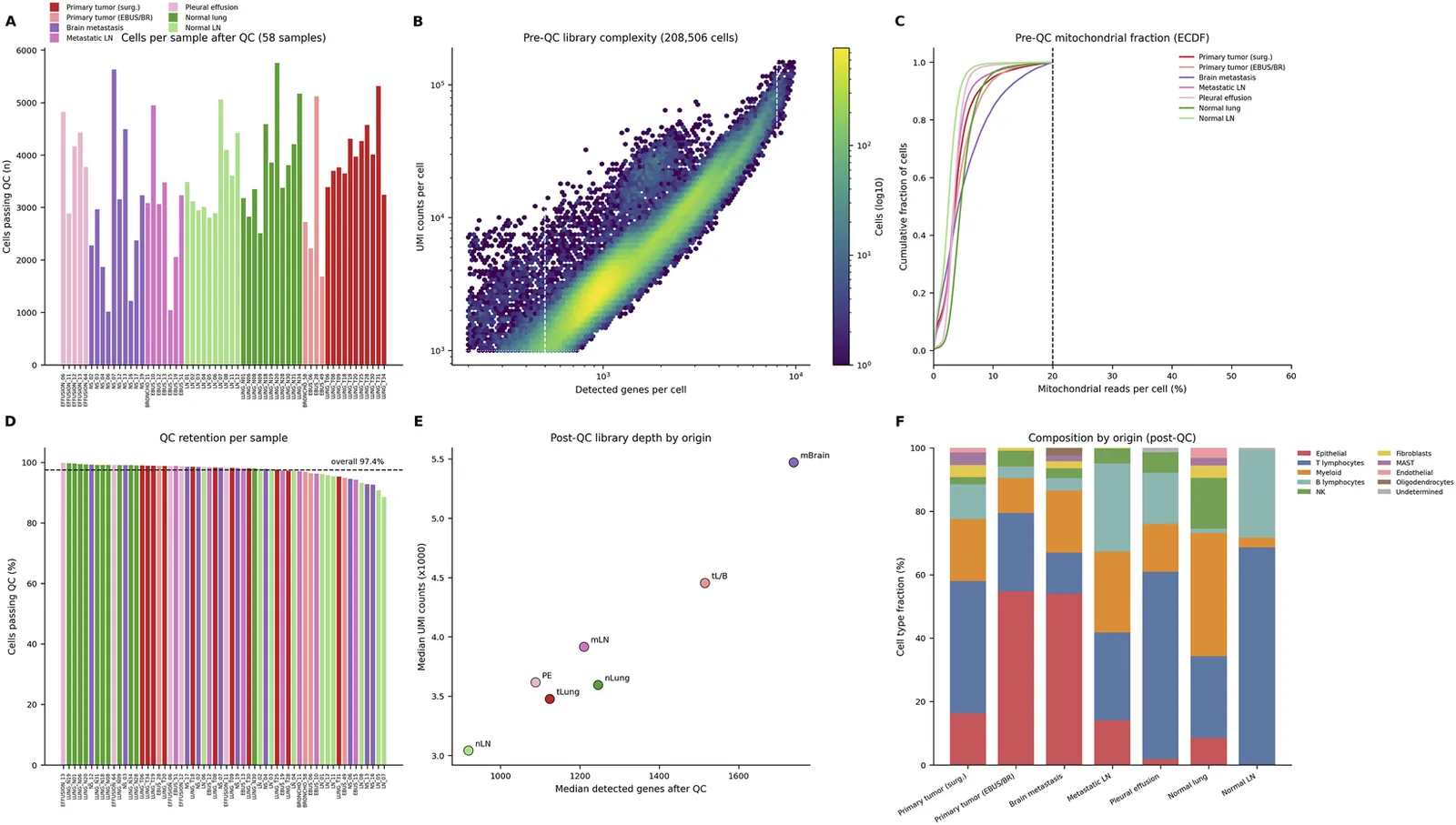
Cohort architecture and quality control of the GSE131907 lung adenocarcinoma atlas. **(A)** Bar plot of QC-passing cells per sample, colored by tissue origin. **(B)** Hexbin of detected genes vs UMI counts before filtering; dashed lines mark the 500–8,000 gene filter. **(C)** ECDF of per-cell mitochondrial read fraction by origin; dashed line = 20% filter. **(D)** QC retention per sample; dashed line marks the overall retention. **(E)** Median detected genes vs median UMIs after QC, one point per origin. **(F)** Stacked fractions of the ten major cell types per origin.

### 3.2 A cellular atlas of lung adenocarcinoma with marker-supported annotations

The retained 203,157 cells were organized by the ten major cell types (Figure 2A) and by tissue origin (Figure 2B), with 40 Leiden transcriptional states (Figure 2C). Canonical markers supported the major-type annotations: epithelial cells expressed EPCAM/KRT8/KRT19, immune compartments expressed CD3D, MS4A1, LYZ/CD68, and NKG7, and stromal compartments expressed COL1A1 and PECAM1 (Figure 2D). Composition shifted across origins: the epithelial fraction was 16.3% in primary tumors versus 8.6% in normal lung, whereas normal lymph nodes were dominated by lymphoid populations (Figure 2E). Refined subtype annotations partitioned each major type into 48 subtypes, the largest being Malignant cells with 24,341 cells (Figure 2F).

**FIGURE 2:**
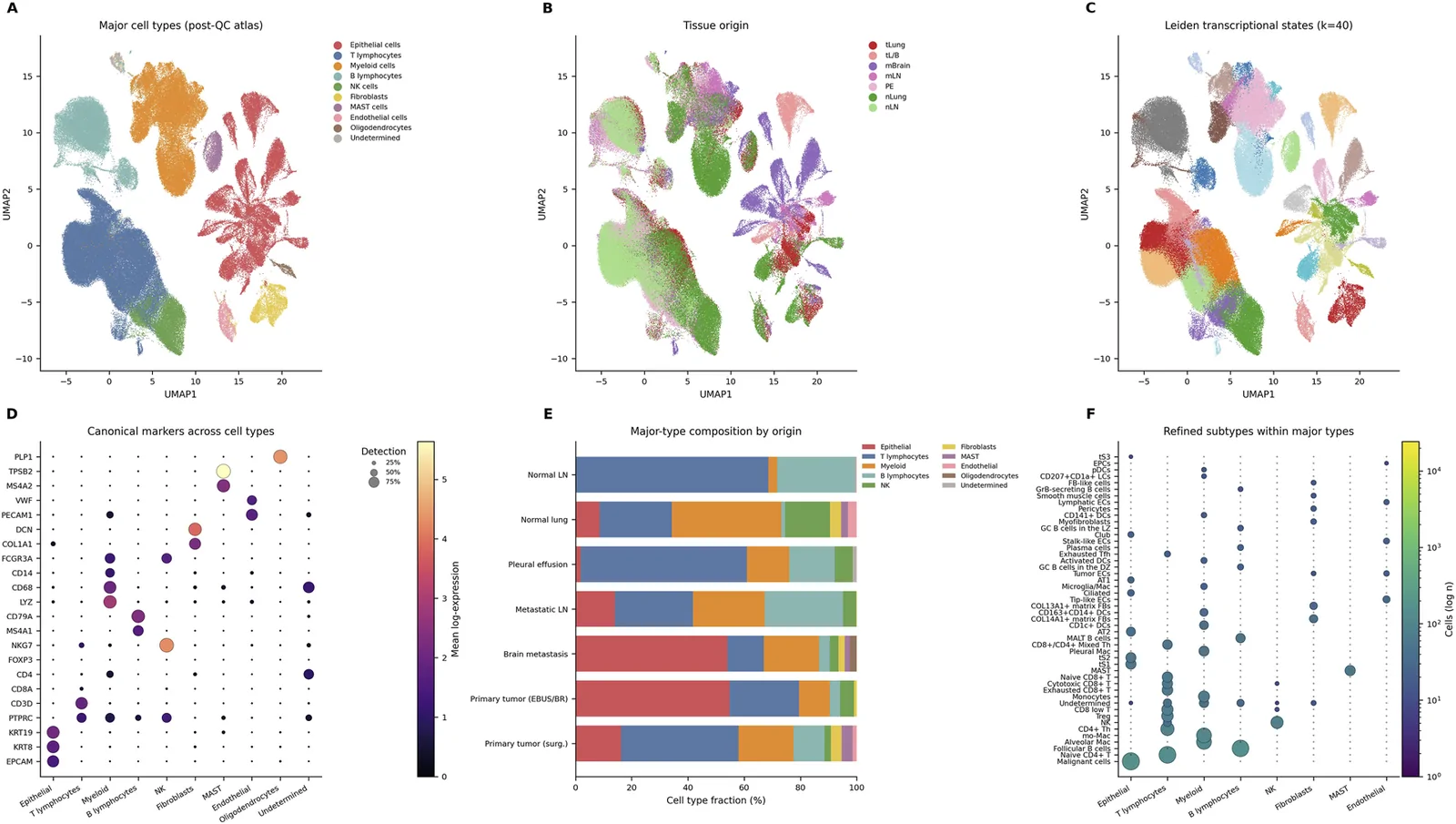
Cellular composition and marker-supported annotation of the single-cell atlas. **(A)** UMAP of all QC-passing cells colored by major cell type. **(B)** UMAP colored by tissue origin. **(C)** UMAP colored by Leiden clusters (resolution 1.0). **(D)** Dot plot of marker mean expression (color) and detection (size) per major type. **(E)** Horizontal stacked composition per origin. **(F)** Bubble matrix of refined subtype abundance (subtypes with >=50 cells).

### 3.3 Malignant epithelial cells resolve into epithelial, hybrid, and mesenchymal states

The malignant compartment contained 24,341 cells. CNV-burden analysis of the sampled cells showed medians of 0.01624 in the 300 malignant-label cells and 0.00515 in the 100 normal epithelial references, a 3.15-fold difference (Supplementary Figure S1A). The 40th/60th-percentile rule classified 4,445 cells as epithelial-like, 3,512 as hybrid E/M, 4,277 as mesenchymal-like, and 12,107 as intermediate (49.7%; Figures 3A–C). The median rule yielded 6,479 epithelial-like, 5,692 hybrid E/M, 6,479 mesenchymal-like, and 5,691 low-E/low-M cells; the quartile rule assigned 19,122 cells (78.6%) to the intermediate category (Supplementary Figure S1B). The hybrid-plus-mesenchymal fraction was 34.7% in brain metastases and 52.9% in metastatic lymph nodes. With gene labels aligned to the expression matrix, mean FN1 log-expression was 0.078, 0.487, and 0.362 in epithelial-like, hybrid E/M, and mesenchymal-like cells, respectively; detection rates were 15.5%, 40.7%, and 29.3% (Figure 3D). VIM mean expression increased from 0.164 in epithelial-like cells to 1.888 in mesenchymal-like cells. Malignant-cell count pseudobulk E and M scores showed ρ = −0.360 (P = 0.109; 21 samples; Figure 3E). The EMT-high versus EMT-low pseudobulk contrast identified 64 genes with BH-adjusted P < 0.05 (28 upregulated and 36 downregulated; Figure 3F).

**FIGURE 3:**
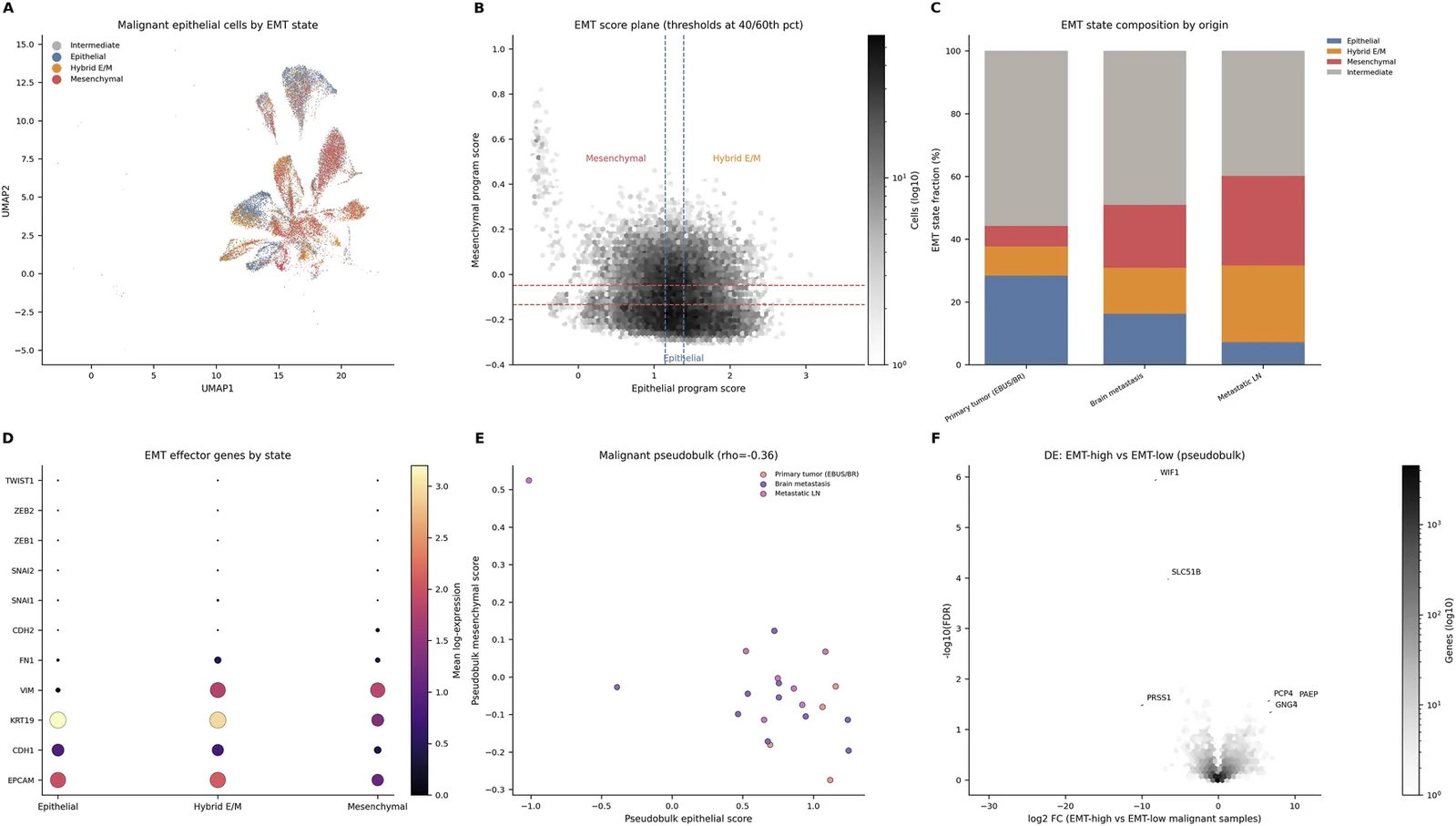
Malignant epithelial EMT states defined on the epithelial/mesenchymal score plane. **(A)** UMAP of malignant epithelial cells colored by EMT state. **(B)** Epithelial versus mesenchymal module scores; dashed lines mark the 40th and 60th percentiles within malignant cells. **(C)** EMT-state fractions by tissue origin. **(D)** Mean log-normalized expression (color) and detection frequency (size) of EMT genes, with expression rows ordered to match the displayed gene labels. **(E)** E and M scores calculated after summing malignant-cell UMI counts within each of 21 samples, followed by normalization and log1p transformation; Spearman ρ = −0.360, P = 0.109. **(F)** Pseudobulk differential expression between EMT-high and EMT-low malignant samples. Threshold sensitivity is shown in Supplementary Figure S1B.

### 3.4 Directional RCD transcriptional profiles vary across malignant EMT states

The median apoptosis balance was −0.153 in epithelial-like cells, −0.103 in hybrid E/M cells, and −0.088 in mesenchymal-like cells. Corresponding ferroptosis balances were −0.428, −0.418, and −0.372 (Figure 4A). Both balances were higher in mesenchymal-like than in epithelial-like cells under the 40th/60th-percentile, median, and quartile rules. The pro- and anti-regulatory components are shown separately for the 21 malignant count-pseudobulk samples (Figure 4B). The DAMP-associated median changed from 0.31 to 0.16 between epithelial-like and mesenchymal-like cells, whereas the inflammasome-associated median changed from −0.14 to −0.15. UMAP displays show the distributions of ferroptosis balance and DAMP-associated transcription (Figures 4C,D), and sample-level correlations are presented in Figure 4E. Gene-level expression differed by component: BIRC3 mean log-expression was 0.54 versus 0.33 and GPX4 was 1.93 versus 1.83 in epithelial-like versus mesenchymal-like cells (Figure 4F). These profiles describe distinct changes in the balance of regulatory transcripts rather than a uniform increase across all RCD programs.

**FIGURE 4:**
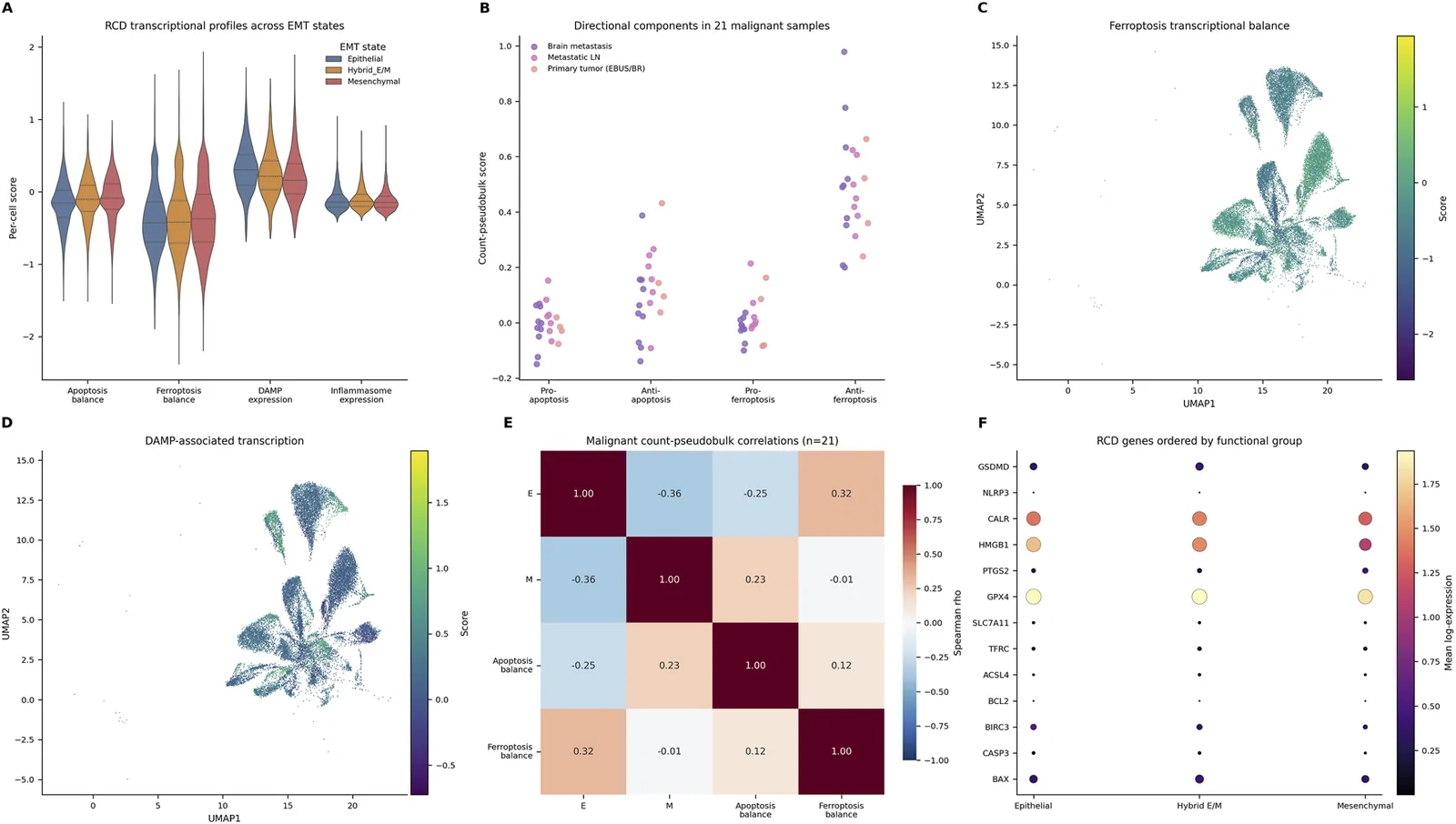
Directional RCD transcriptional profiles across malignant EMT states. **(A)** Per-cell distributions of apoptosis balance, ferroptosis balance, DAMP-associated expression, and inflammasome-associated expression. **(B)** Separate pro-/anti-apoptosis and pro-/anti-ferroptosis scores in 21 malignant count-pseudobulk samples, colored by tissue origin. **(C)** Malignant-cell UMAP colored by ferroptosis balance. **(D)** UMAP colored by DAMP-associated expression. **(E)** Spearman correlations among E, M, apoptosis-balance, and ferroptosis-balance scores across the 21 malignant pseudobulk samples. **(F)** Gene-level mean expression (color) and detection frequency (size), ordered by functional group. Balance indices equal the pro-regulatory score minus the anti-regulatory score; PTGS2 is displayed as a separate response marker.

### 3.5 The immune microenvironment varies by tissue origin and tracks malignant EMT state

Immune cells (156,721) resolved into the expected lymphoid and myeloid compartments; the five most abundant official subtypes were Naive CD4^+^ T, Follicular B cells, Alveolar Mac, mo-Mac, CD4^+^ Th (Figure 5A; composition by origin in Figure 5B, where the dominant subtype in primary tumors was mo-Mac). Per-sample T/NK effector and exhaustion scores were not significantly associated (Spearman rho = 0.23, p = 0.078) across samples (Figure 5C), and marker evidence supported the subtype annotations (Figure 5D). Exhausted T/NK cells occupied distinct UMAP regions (89449 T/NK cells; Figure 5E). Across the 21 malignant-cell samples, the malignant EMT-high fraction was positively associated (Spearman rho = 0.54, P = 0.012; BH-adjusted q = 0.059 across five cellular-fraction comparisons) with the myeloid fraction, whereas the T-cell (rho = 0.04), NK (rho = −0.02), B-cell (rho = 0.13), and fibroblast (rho = 0.15) fractions were not significant (Figure 5F).

**FIGURE 5:**
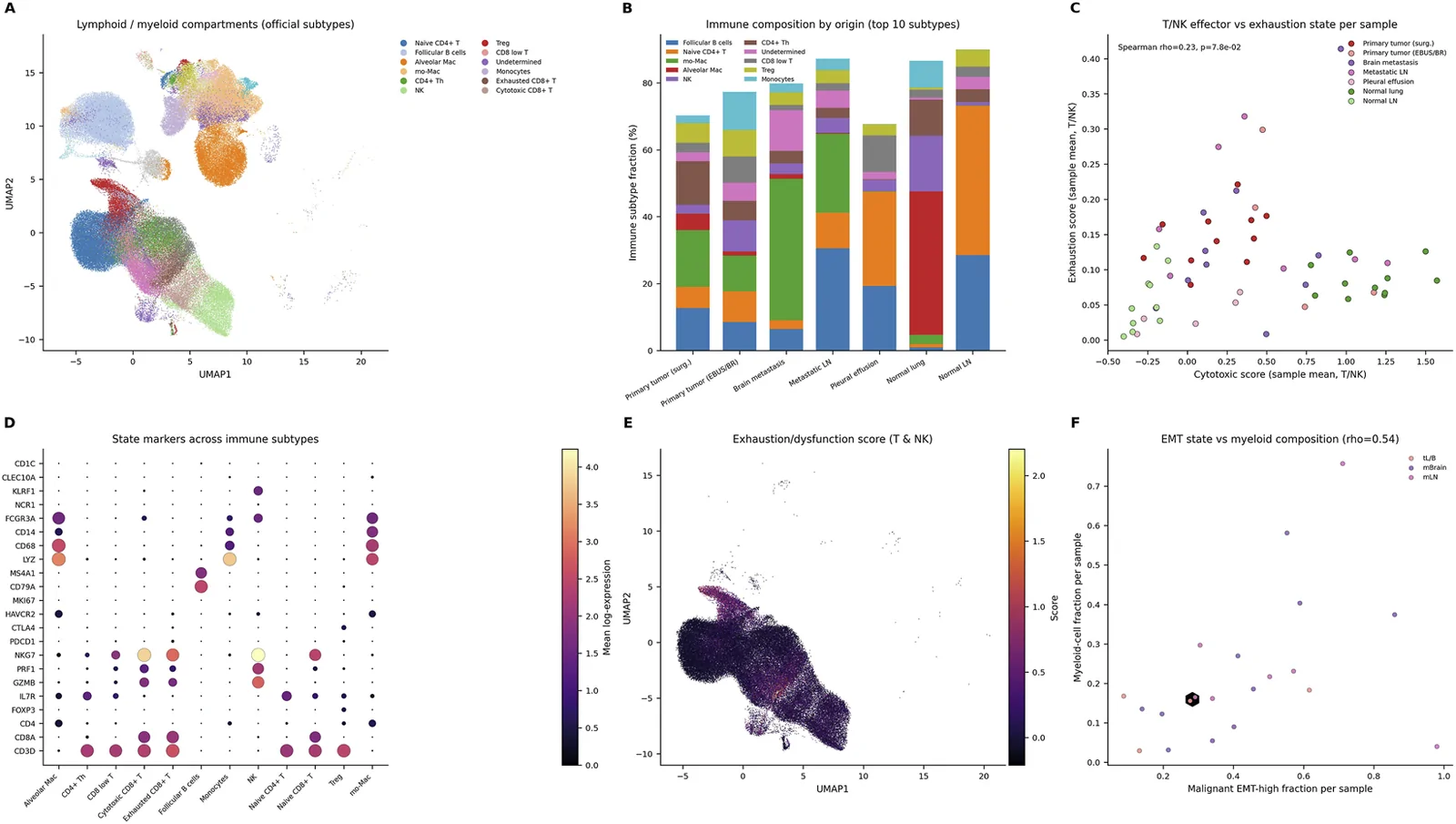
Immune microenvironment composition and its association with malignant EMT state. **(A)** UMAP of immune cells colored by official subtype annotation (top 12 types in legend). **(B)** Top-10 refined immune subtype fractions per origin. **(C)** Per-sample mean cytotoxic vs exhaustion scores among T/NK cells. **(D)** Marker mean expression (color) and detection (size) across top-12 immune subtypes. **(E)** UMAP of T/NK cells colored by exhaustion score. **(F)** Per-sample malignant EMT-high fraction vs myeloid fraction; Spearman statistics computed at the sample level (n = 21). The myeloid association has P = 0.012 and BH-adjusted q = 0.059 across the five cellular-fraction comparisons.

### 3.6 Malignant-to-immune signaling shifts with EMT sender state

Across the curated ligand-receptor proxy, the strongest malignant-to-immune pathways included MIF_CD74 (mean proxy score 1.82; Figure 6A). Contrasting sender states, 8 of 12 pathways scored higher for EMT-high than for epithelial-state senders (Figure 6B). Ligand expression shifted with state: HMGB1 mean log-expression was 1.09 in mesenchymal-like versus 1.68 in epithelial-like senders (Figure 6C). Receptors were differentially distributed across immune compartments (18 receptors × 8 receivers; Figure 6D). The largest sender-state differences involved VIM_CD44 toward Alveolar Mac (delta = 1.30; Figure 6E), and DAMP-sensing competence (AGER/TLR4/LRP1) was highest in CD1c+ DCs (Figure 6F).

**FIGURE 6:**
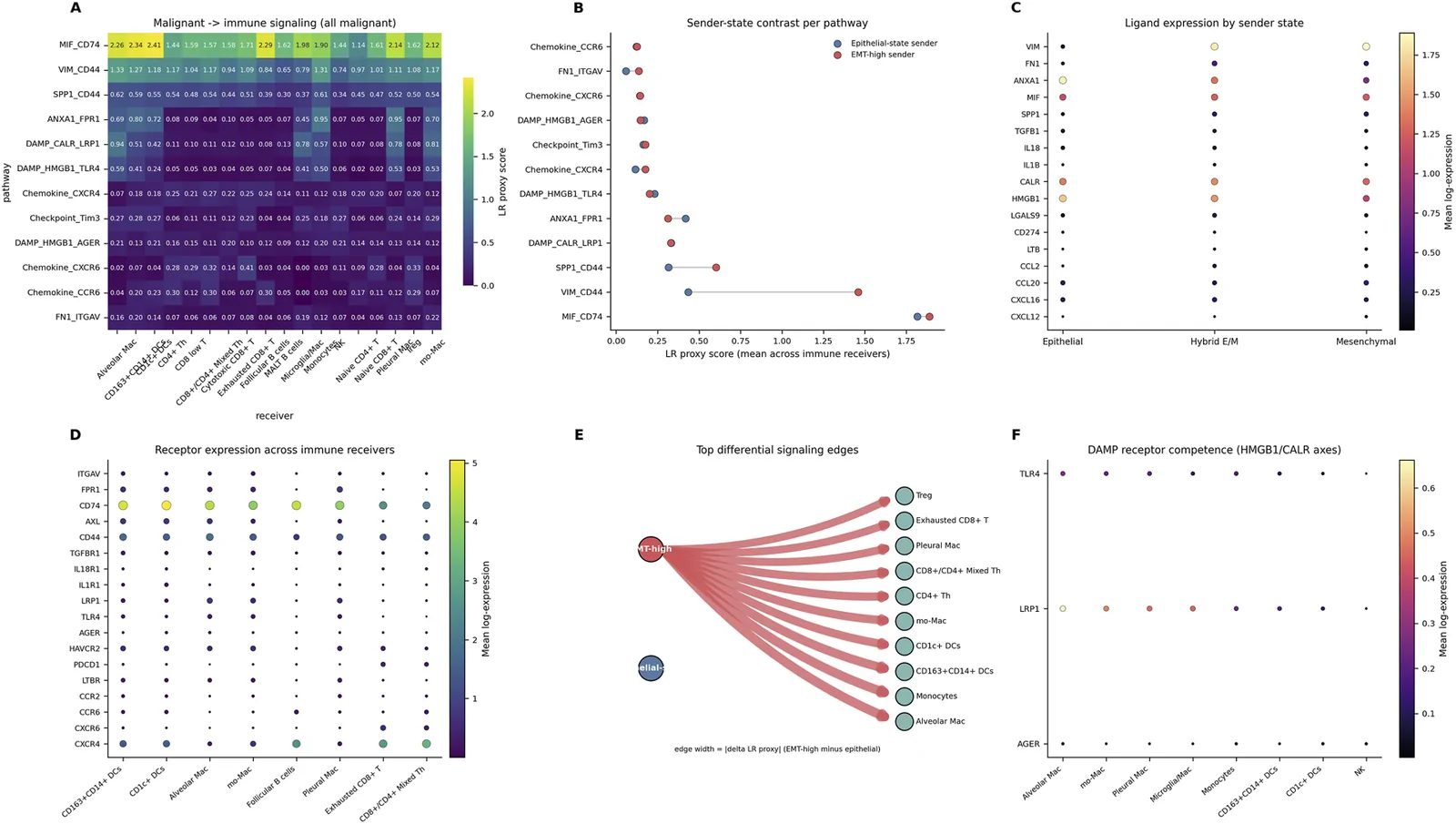
Malignant-to-immune ligand-receptor signaling proxy by EMT sender state. **(A)** Mean-malignant sender LR proxy scores across immune receivers (top 12 pathways). **(B)** Mean LR proxy per pathway for EMT-high vs epithelial senders. **(C)** Ligand mean expression (color, size) per sender state. **(D)** Receptor mean expression (color, size) across immune receiver types. **(E)** Top-10 edges by |delta|; red = higher in EMT-high sender, blue = lower; no self-loops. **(F)** Receptor expression for the DAMP axes (AGER/TLR4/LRP1) across immune receivers.

### 3.7 Cross-platform tumor-versus-normal expression concordance in TCGA-LUAD

In TCGA-LUAD, 539 tumors and 59 normal samples separated in the sample-sample correlation structure (Figure 7A). Relative to normal samples, tumors showed higher EPCAM (log2 fold change = 1.61), CDH1 (1.00), BIRC3 (0.39), and GPX4 (0.29), and lower VIM (−1.15), all with BH-adjusted P < 0.05. FN1 (log2 fold change = −0.045, adjusted P = 0.849) and CXCR4 (−0.003, adjusted P = 0.986) showed little tumor-versus-normal difference (Figures 7B,C). Differential expression identified 13,707 significant genes among 19,511 tested. Across 17,124 shared genes, direction concordance was 68.8% overall and 91.5% among genes significant in both platforms, with Spearman ρ = 0.50 and 0.82, respectively (Figures 7D,E). BIRC3 and CXCR4 expression correlated across tumors (ρ = 0.43; Figure 7F). The composite and the EMT-only index were strongly correlated across the 539 tumors (Spearman ρ = 0.982; Supplementary Table S6).

**FIGURE 7:**
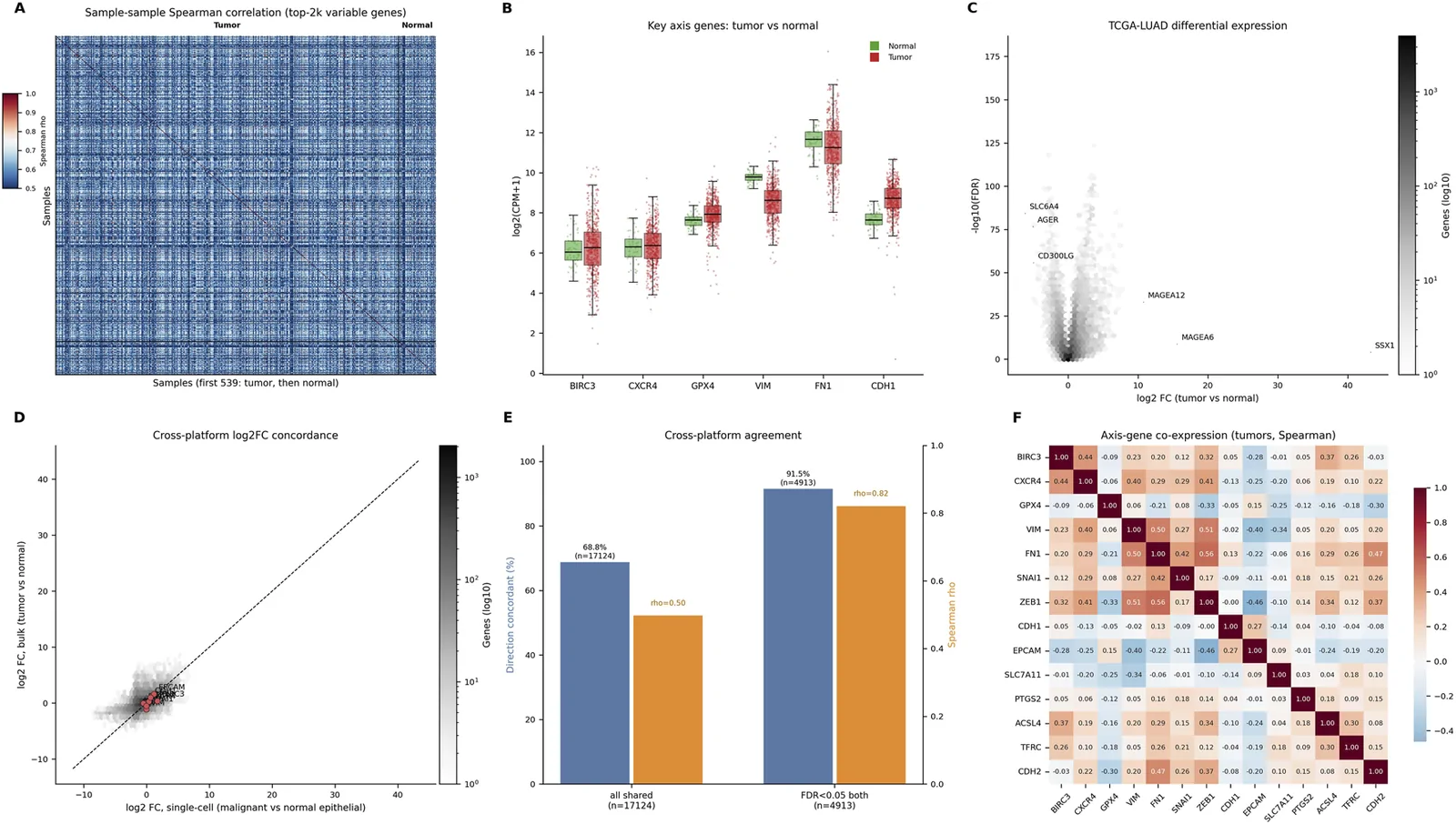
TCGA-LUAD tumor-versus-normal differential expression and cross-platform concordance. **(A)** Sample-sample Spearman correlation on top-2,000 variable genes; axis verified samples x samples. **(B)** log2(CPM+1) per sample for key genes; box + individual samples. **(C)** PyDESeq2 tumor-vs-normal volcano; top |log2FC| labeled. **(D)** sc malignant-vs-normal-epithelial vs. bulk tumor-vs-normal log2FC; key genes highlighted. **(E)** Direction concordance and Spearman rho, all shared vs. FDR < 0.05 genes. **(F)** Tumor-sample Spearman correlations among axis genes.

### 3.8 Stage dominates survival; the EMT-RCD score is not an independent risk factor in TCGA-LUAD

Among 507 patients with follow-up, the score-high group had 74 deaths among 245 patients and the score-low group had 109 among 262 (log-rank P = 0.070; Figure 8A). The corresponding log-rank P values were 0.064 for BIRC3, 0.210 for CXCR4, and 0.033 for GPX4 (Figures 8B,C). In the 478-patient complete-case Cox model, the composite score had HR = 0.973 (95% CI 0.829–1.141; P = 0.736), whereas stage IV versus I had HR = 3.68 (P < 0.001; Figure 8D). The component models gave HR = 0.993 for the EMT axis (95% CI 0.860–1.147; P = 0.928), 1.086 for apoptosis balance (0.837–1.410; P = 0.536), 1.032 for ferroptosis balance (0.898–1.187; P = 0.658), and 1.018 for their directional sum (0.935–1.108; P = 0.677; Supplementary Figure S1C; Supplementary Table S6). The composite score did not differ significantly across stages (Kruskal–Wallis P = 0.236) and was lower in deceased than living patients at last follow-up (medians −0.32 versus −0.04; Mann–Whitney P = 0.033; Figures 8E,F). The time-to-event models showed no independent survival association for the composite or directional scores.

**FIGURE 8:**
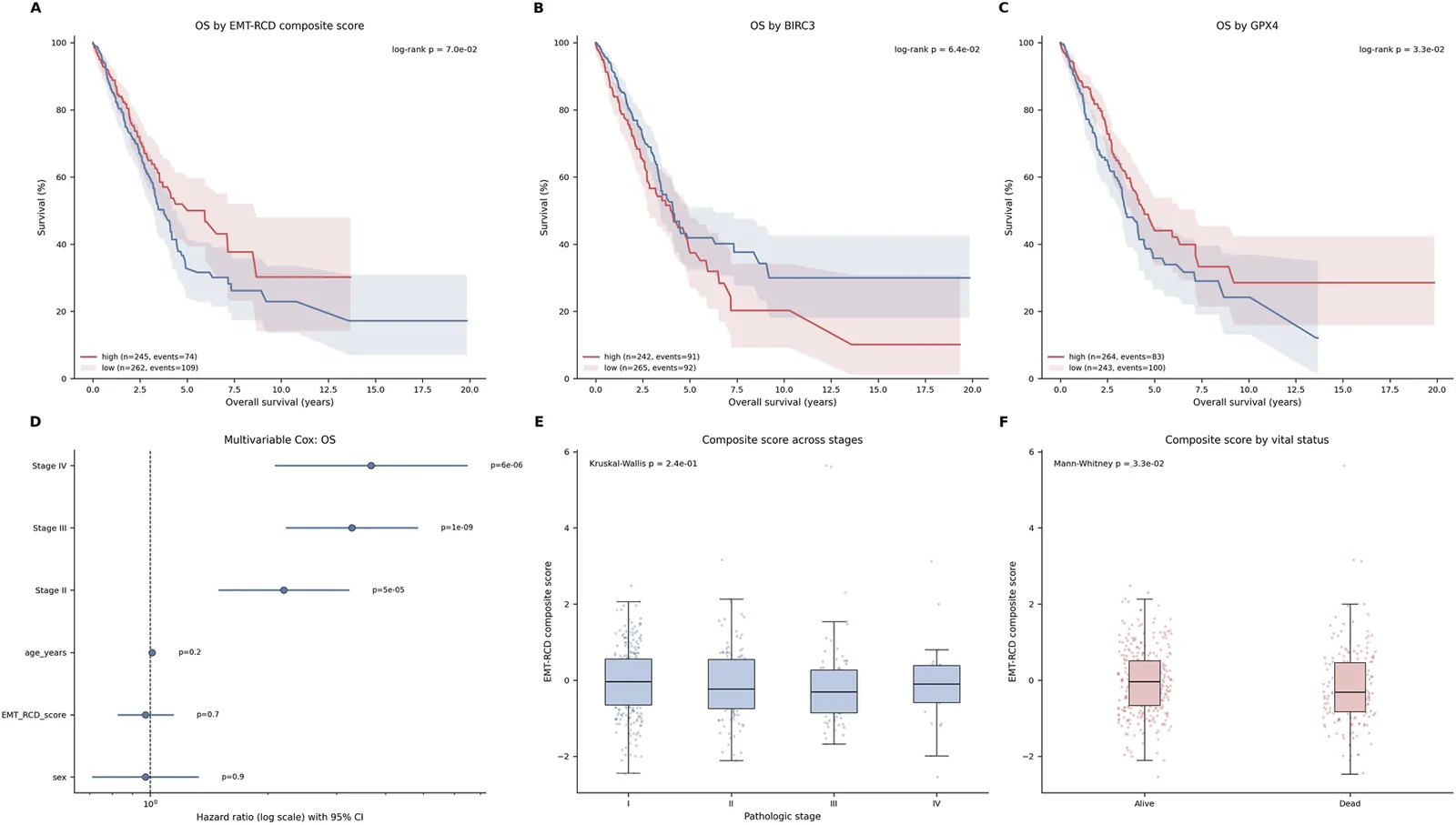
EMT–RCD composite transcriptional score and overall survival in TCGA-LUAD. **(A)** Kaplan–Meier overall survival with 95% confidence bands, grouped by the composite-score median of the 539-tumor expression cohort; 507 patients have survival follow-up. **(B,C)** Kaplan–Meier survival by BIRC3 and GPX4 expression. **(D)** Multivariable Cox model adjusted for age, sex, and stage, using 478 complete cases and 172 deaths. **(E)** Composite score by pathologic stage. **(F)** Composite score by vital status at last follow-up. Directional component Cox analyses are shown in Supplementary Figure S1C.

### 3.9 RT-qPCR analysis of gene expression in A549 and NCI-H1975 cells

RT-qPCR revealed cell-line-dependent expression responses following TGF-β1 treatment (10 ng/mL, 48 h) (Figure 9). In A549 cells, BIRC3 increased 1.34-fold (raw P = 0.0292, BH-adjusted P = 0.0390), CXCR4 increased 2.43-fold (raw P = 0.00045, BH-adjusted P = 0.0018), and MALAT1 increased 1.63-fold (raw P = 0.00281, BH-adjusted P = 0.0056) relative to controls. GPX4 was 0.75-fold relative to controls (raw P = 0.1885, BH-adjusted P = 0.1885). In NCI-H1975 cells, CXCR4 increased 2.12-fold (raw P = 0.00467, BH-adjusted P = 0.0093) and MALAT1 increased 1.33-fold (raw P = 0.1696, BH-adjusted P = 0.1696) in the supplied matrix; BIRC3 and GPX4 changes remain reported from the source summary because their Cq values were not supplied. CXCR4 was therefore the consistent expression response across the two cell lines. The concordant CXCR4 response to this EMT-inducing stimulus provides a gene-level link to the cell-state programs examined in the transcriptomic analyses.

**FIGURE 9:**
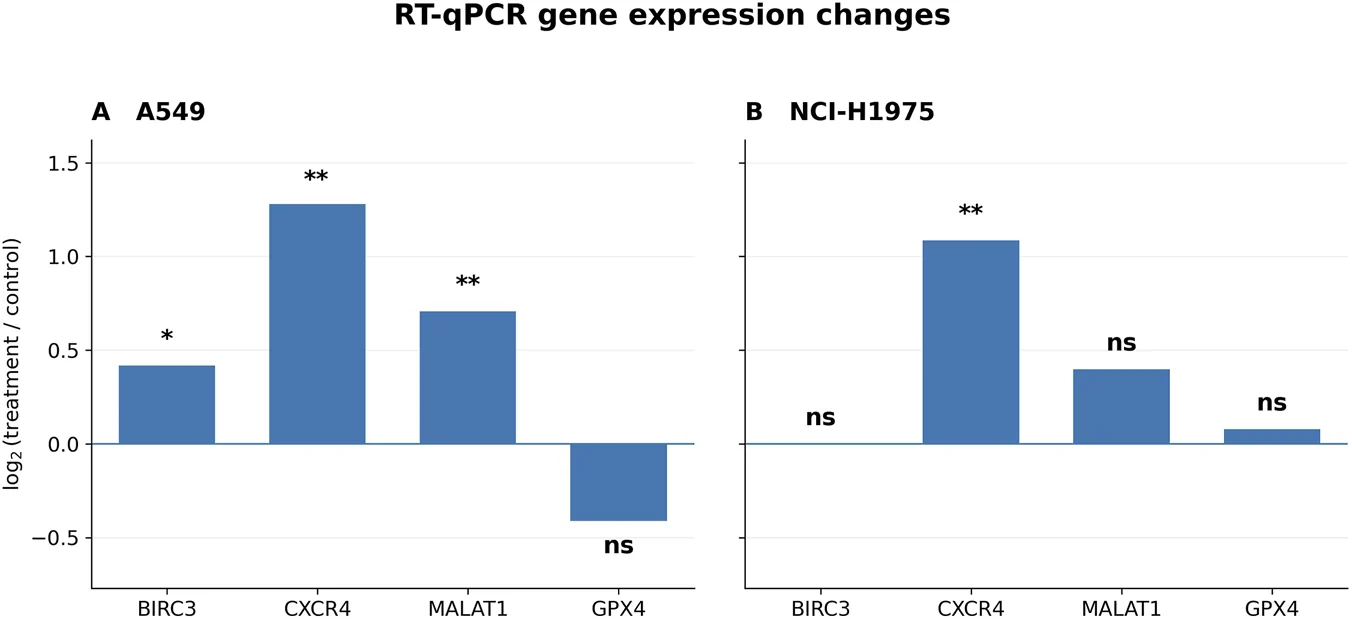
RT-qPCR expression changes after TGF-β1 treatment in A549 and NCI-H1975 cells. **(A)** Relative expression changes of BIRC3, CXCR4, MALAT1, and GPX4 in A549 cells. **(B)** Relative expression changes of the same four genes in NCI-H1975 cells. Bars represent log2 fold changes (log2FC), calculated as the base-2 logarithm of the ratio of mean normalized expression in the treated group to that in the control group. Positive and negative values indicate increased and decreased expression, respectively. Each group comprised four biological replicates (n = 4), with three technical replicates per biological replicate. P values were adjusted using the Benjamini–Hochberg method across the genes with available biological-replicate Cq values within each cell line (four genes in A549 and two in NCI‐H1975). For these genes, significance is shown using adjusted P values: *P < 0.05 and **P < 0.01; ns, not significant. The significance labels for BIRC3 and GPX4 in NCI‐H1975 cells were taken from the source experimental summary because their biological‐replicate Cq values were unavailable.

## 4 Discussion

The joint analysis identifies EMT-associated transcriptional heterogeneity in LUAD and connects it with variation in death-regulatory expression and immune composition. The tumor-versus-normal comparisons showed substantial cross-platform agreement among differentially expressed genes, while single-cell resolution identified state-dependent patterns within the malignant compartment. The positive sample-level association between the EMT-high fraction and myeloid abundance points to a connection between tumor-cell state and the surrounding immune population. The expression-based CNV comparison adds a separate genomic-pattern assessment to the marker-based evaluation of the malignant compartment.

Separating pro- and anti-regulatory genes clarifies the interpretation of RCD expression. The higher pro-minus-anti balances in mesenchymal-like cells reflect relative changes among the selected regulatory transcripts. In contrast, the DAMP-associated program was lower and the inflammasome-associated program changed little, indicating that the programs differ in both direction and magnitude. GPX4 and SLC7A11 represent ferroptosis defense, whereas ACSL4 and TFRC represent lipid-remodeling and iron-uptake components associated with ferroptosis susceptibility (Jiang et al., 2021; Viswanathan et al., 2017; Hangauer et al., 2017; Chen et al., 2021). PTGS2 is displayed separately because its induction was identified as a downstream marker in the GPX4 study (Yang et al., 2014). This gene-resolved analysis provides a biological interpretation for the differences observed across cell states.

Epithelial and mesenchymal programs were analyzed as two dimensions, consistent with the coexistence of epithelial, hybrid, and mesenchymal features described in EMT research (Nieto et al., 2016; Yang et al., 2020). Under the 40th/60th-percentile rule, the intermediate group includes central-band cells and low-E/low-M cells. Its size therefore depends on the classification boundaries: it represented 49.7% of malignant cells under the primary rule, 23.4% under the median rule, and 78.6% under the quartile rule. The direction of the epithelial-like versus mesenchymal-like RCD-balance comparison remained the same under all three rules. The modest E/M correlation across malignant pseudobulk samples summarizes sample-level co-variation and addresses a different question from the two-dimensional state assignment of individual cells.

The composite score and the cell-line experiment assess complementary outcomes. The composite summarizes mesenchymal, survival-regulatory, chemokine, and ferroptosis-defense expression in tumors, while RT-qPCR quantifies the response of individual candidate genes within each cell line. CXCR4 increased in both A549 and NCI-H1975 cells, making it the most consistent response among the four tested genes. Published NSCLC experiments connect CXCR4 with STAT3/Slug signaling and radioresistance (Kim et al., 2021), providing a biological context for this finding. CXCR4 increased following TGF-β1 exposure in both tested cell lines, consistent with a response to an EMT-inducing stimulus. Its response to treatment can coexist with little tumor-versus-normal difference in TCGA and with a null overall-survival association, because these comparisons concern different biological contrasts.

The composition of the score provides context for the numerically more favorable survival of the composite-score-high group. Its positive term contains BIRC3 and GPX4, which represent cell-death regulation and defense, as well as CXCR4 and mesenchymal genes; increasing this expression summary is therefore not equivalent to increasing all pro-death programs. In addition, a cell-state expression pattern does not determine a fixed direction of overall-survival association across patients. The score-high versus score-low log-rank P value was 0.070, and the adjusted hazard ratio was close to 1. Separate EMT, apoptosis-balance, and ferroptosis-balance analyses gave the same null survival conclusion. Thus, the principal finding is the heterogeneity of transcriptional programs and their immune associations, while pathologic stage remained the dominant survival-associated variable.

BIRC3/IAP, CXCR4, and GPX4 biology provides complementary routes for studying how tumor-cell state interacts with stress tolerance and immune responses (Viswanathan et al., 2017; Hangauer et al., 2017; Kim et al., 2021; Salvesen and Duckett, 2002; Fulda, 2017; Chen et al., 2021; Ribas and Wolchok, 2018; Bagchi et al., 2021). The consistent CXCR4 response across the two cell lines supports prioritizing this pathway in subsequent studies of state-dependent gene regulation.

## 5 Conclusion

LUAD malignant cells show heterogeneous epithelial and mesenchymal transcription accompanied by distinct RCD-regulatory and DAMP-associated profiles. The EMT-high fraction correlated positively with myeloid abundance, and tumor-versus-normal gene-expression changes agreed across single-cell and bulk datasets. CXCR4 increased consistently in both tested cell lines. The EMT–RCD composite and directional component scores showed no independent overall-survival association, placing the main biological emphasis on cell-state variation and CXCR4-associated expression.

## Data Availability

The datasets presented in this study can be found in online repositories. The names of the repository/repositories and accession number(s) can be found in the article/Supplementary Material.

## Supplementary material

SUPPLEMENTARY FIGURE S1 CNV assessment, EMT-threshold sensitivity, and directional survival analyses. **(A)** Inferred CNV-burden distributions in 300 malignant-label cells and 100 normal epithelial reference cells; the respective medians are 0.01624 and 0.00515. **(B)** Four-category assignments under the 40th/60th-percentile, median, and 25th/75th-percentile rules using the same primary E/M scores. Under the median rule, the intermediate category consists of low-E/low-M cells. **(C)** Continuous-score Cox models adjusted for age, sex, and stage in 478 patients with 172 deaths. Points and intervals represent hazard ratios and 95% confidence intervals.
